# Circulating Proteins as Predictors of Improvement in Physical Function in Hospitalized Older Adults with Geriatric Syndromes: Findings from the REHAB-HF Trial

**DOI:** 10.64898/2026.09.22.26363692

**Published:** 2026-09-23

**Authors:** Abdulla A. Damluji, Scott A. Bruce, Christopher R. deFilippi

## Abstract

**Introduction:**

Circulating proteins may augment prognostic scores and provide mechanistic insights for cardiovascular disease. Their role for adding precision to predicting response to therapeutic exercise training is less certain. Older adults with HF frequently experience geriatric syndromes, which may influence response to a rehabilitation intervention (RI). We sought to determine if the functional benefits of a 12-week tailored, structured, and progressive multidomain physical RI program in older adults, following a HF admission, could be estimated by pre-intervention levels of proteins.

**Methods:**

Hospitalized adults age ≥ 60 years with HF were randomized to 12-week multidimensional (aerobic, strength, balance, flexibility) physical therapist-led RI versus attention control (AC). Outcomes included change in the short physical performance battery (SPPB) and 6-minute walk distance (6MWD). At pre-intervention and 12-weeks, 92 circulating proteins associated with CVD and inflammation were measured. Using linear regression, LASSO and ensemble tree-based approaches for flexible feature selection, proteins were selected for stratification of treatment response. Decision trees were developed to incorporate a multi-protein approach to optimize stratification of treatment response (effect measure modifiers [EMM]) for both functional outcomes. Differences in 12-week temporal changes based on RI vs AC and mediation analysis were used to assess possible causal roles for these proteins.

**Results:**

Baseline proteomic data was available in 243/349 participants (69.6%). For SPPB, 2 proteins (GP6, ST2), and for 6MWD, 3 proteins (ALCAM, Gal-4, PECAM-1) were selected as EMMs. For SPPB, a decision tree with ST2 followed by GP6 level identified a differential 12-week improvement in score for RI vs AC from −0.8 (95%CI −1.9, 0.2) to 2.1 (95%CI 1.5, 2.7). For 6MWD, the optimized decision tree was based only on ALCAM with a differential 12-week improvement in score for RI vs AC from 10m (95%CI −17m, 36m) to 85m (95%CI 56m, 113m). However, none of the EMM proteins were mediators of change for the functional outcomes.

**Conclusions:**

Circulating proteins related to CVD and inflammation (GP6, ST2, ALCAM) stratify a successful functional response to the REHAB-HF rehabilitation program in older adults following HF hospitalization. Further investigation is needed to identify proteins associated with a causal role for rehabilitation benefits.

## Full Text Availability

The license terms selected by the author(s) for this preprint version do not permit archiving in PMC. The full text is available from the preprint server.

